# Protein lactylation in Alzheimer’s disease: bridging metabolism, pathology, and therapeutic opportunity

**DOI:** 10.3389/fnagi.2026.1790090

**Published:** 2026-03-20

**Authors:** Huawen Cao, Junyi Liang, Xiaohong Dong, Zhiqi Xia, Xiaoting Luo, Bin Liu

**Affiliations:** 1 Heilongjiang University of Traditional Chinese Medicine, Harbin, Heilongjiang, China; 2 Heilongjiang University of Traditional Chinese Medicine, Jiamusi College, Jiamusi, Heilongjiang, China

**Keywords:** Alzheimer’s disease, cell-specific mechanisms, epigenetic control, lactate metabolism, lactylation pathways, metabolic reprogramming

## Abstract

Lactate, long regarded as a mere by-product of glycolysis, is increasingly recognized as a signaling metabolite and epigenetic regulator through protein lactylation. This lysine-specific post-translational modification functionally couples cellular metabolic states to gene regulatory programs and orchestrates cell type–specific functions across neurons, astrocytes, and microglia, thereby shaping synaptic plasticity, neuroinflammatory responses, and protein aggregation. Accumulating evidence implicates dysregulated lactylation in the pathogenesis of Alzheimer’s disease (AD), where it modulates amyloid-β deposition, tau aggregation, and glial reactivity. In this Review, we summarize the enzymatic regulation of protein lactylation, delineate its context-dependent roles in distinct central nervous system cell types, and highlight its function as a metabolic–epigenetic–immune nexus in AD progression. We further discuss emerging therapeutic strategies targeting lactate metabolism and lactylation pathways, and outline critical knowledge gaps that must be addressed to translate these insights into innovative diagnostic and therapeutic approaches. By integrating metabolic reprogramming, epigenetic control, and cell-specific mechanisms, this Review positions lactylation as a compelling and emerging frontier in AD research.

## 1 Introduction

Protein lactylation, an emerging metabolic–epigenetic coupling mechanism, has rapidly advanced to the forefront of biomedical research. Lactate, acting both as a modification donor and a pivotal regulatory signaling molecule, plays an integral role in the modulation of brain function, thereby opening new therapeutic avenues for a broad spectrum of neurological disorders (Chen Y. et al., 2025b). Notably, aberrant lactylation driven by metabolic dysfunction has been increasingly implicated in the initiation and progression of multiple neurological diseases (Gu et al., 2026). Alzheimer’s disease (AD), the most prevalent form of dementia, is characterized by progressive memory impairment and cognitive decline (Tzioras et al., 2023). With global population aging, the incidence of AD is projected to double by 2050 (Alzheimers Dement, 2025). Despite decades of intensive investigation, effective interventions capable of halting or reversing disease progression remain elusive. Beyond the classical pathological hallmarks—amyloid-β (Aβ) plaques, tau hyperphosphorylation, and chronic neuroinflammation—emerging evidence underscores metabolic dysregulation, particularly impaired cerebral glucose utilization, as an early and central driver of neuronal dysfunction (Butterfield and Halliwell, 2019; Tzioras et al., 2023). Importantly, cerebral hypometabolism frequently precedes overt neurodegeneration and cognitive deficits, indicating that metabolic alterations are not merely secondary consequences but rather constitute critical upstream events in AD pathogenesis (Terstege et al., 2024).

Among metabolic intermediates, lactate has undergone a conceptual transformation from a glycolytic by-product to a multifunctional signaling metabolite and epigenetic regulator (Ferguson et al., 2018). The identification of protein lactylation as a post-translational modification established a direct mechanistic connection between cellular metabolism and gene regulation (Zhang et al., 2019). Subsequent studies have demonstrated that lactylation is not confined to histones—where it modulates transcription through chromatin remodeling—but is also widespread on non-histone proteins, where it directly influences protein activity and function across diverse physiological and pathological contexts (Gu et al., 2022; Shi P. et al., 2025). Protein lactylation thus establishes a critical metabolic–transcriptional interface and exerts cell type–specific effects in neurons, astrocytes, and microglia. Growing evidence indicates that dysregulated lactylation promotes AD pathogenesis by modulating Aβ deposition, tau aggregation, neuroinflammatory responses, and oxidative stress (Li et al., 2022). In this Review, we focus on the enzymatic regulators of lactylation—including writers, erasers, and readers—their cell-type-specific roles in AD, and the function of lactylation as a metabolic–epigenetic–immune hub. We further discuss its potential as a therapeutic target and highlight key knowledge gaps that must be addressed to advance our understanding and treatment of AD.

## 2 Lactate in the AD brain

In the brain, lactate is primarily generated from glucose through glycolysis. Under physiological conditions, the metabolic division of labor between neurons and glial cells—particularly astrocytes—constitutes the canonical astrocyte–neuron lactate shuttle (ANLS) model (Wu et al., 2023). Lactate is exported from astrocytes into the extracellular space via members of the monocarboxylate transporter (MCT) family, predominantly MCT1 and MCT4, and is subsequently taken up by neurons through the highly expressed neuronal transporter MCT2 (Yang C. et al., 2024). Within neurons, lactate is converted to pyruvate by lactate dehydrogenase 1 (LDH1), enters the tricarboxylic acid cycle, and undergoes efficient oxidative metabolism to sustain synaptic activity and neuronal signaling (Yang C. et al., 2024). This shuttle mechanism highlights lactate as a critical intercellular “energy currency,” enabling metabolic coupling and resource reutilization across cell types.

Intracellular lactate follows two principal metabolic fates: mitochondrial oxidation, as described above, or utilization as a direct precursor for protein lactylation. For lactylation to occur, lactate must first be converted into its activated form, lactyl–coenzyme A (Zhang et al., 2019). Emerging evidence suggests that lactyl–CoA may be generated directly through the catalytic activity of GTP-specific succinyl-CoA synthetase (GTP-SCS) and acyl-CoA synthetase short-chain family member 2 (ACSS2) (Liu R. et al., 2025; Zhu et al., 2025). This conversion establishes a direct biochemical conduit between metabolic flux and the epigenetic modification machinery, enabling fluctuations in lactate abundance and metabolic throughput to be sensed and transduced into covalent protein modification signals.

In the early stages of AD, and often preceding overt clinical symptoms, the brain exhibits characteristic reductions in glucose metabolism and region-specific energetic insufficiency (An et al., 2018; Huang et al., 2023). During this phase, cerebrospinal fluid (CSF) lactate levels may rise concomitantly with declining cerebral glucose utilization, particularly in regions associated with the default mode network (DMN) (Liguori et al., 2016). As a compensatory or maladaptive response to mitochondrial dysfunction, glycolytic pathways are frequently aberrantly activated, giving rise to a state reminiscent of aerobic glycolysis (the Warburg effect) (Miao et al., 2023; Minhas et al., 2024). Although this shift may initially mitigate neuronal energy deficits, it ultimately promotes lactate accumulation and exacerbates neuronal dysfunction (Calì et al., 2024). Concurrently, impaired mitochondrial function may compromise lactate clearance, further amplifying its accumulation (Morello et al., 2023). The combined effects of increased production and reduced elimination result in abnormally elevated lactate concentrations within local microenvironments, such as the vicinity of Aβ plaques. This pathological lactate buildup not only perturbs cellular function through microenvironmental acidification but, more critically, supplies excess substrate for aberrant protein lactylation, thereby converting metabolic disturbances into persistent epigenetic and functional dysregulation (Zhang et al., 2019).

**Figure 1:**
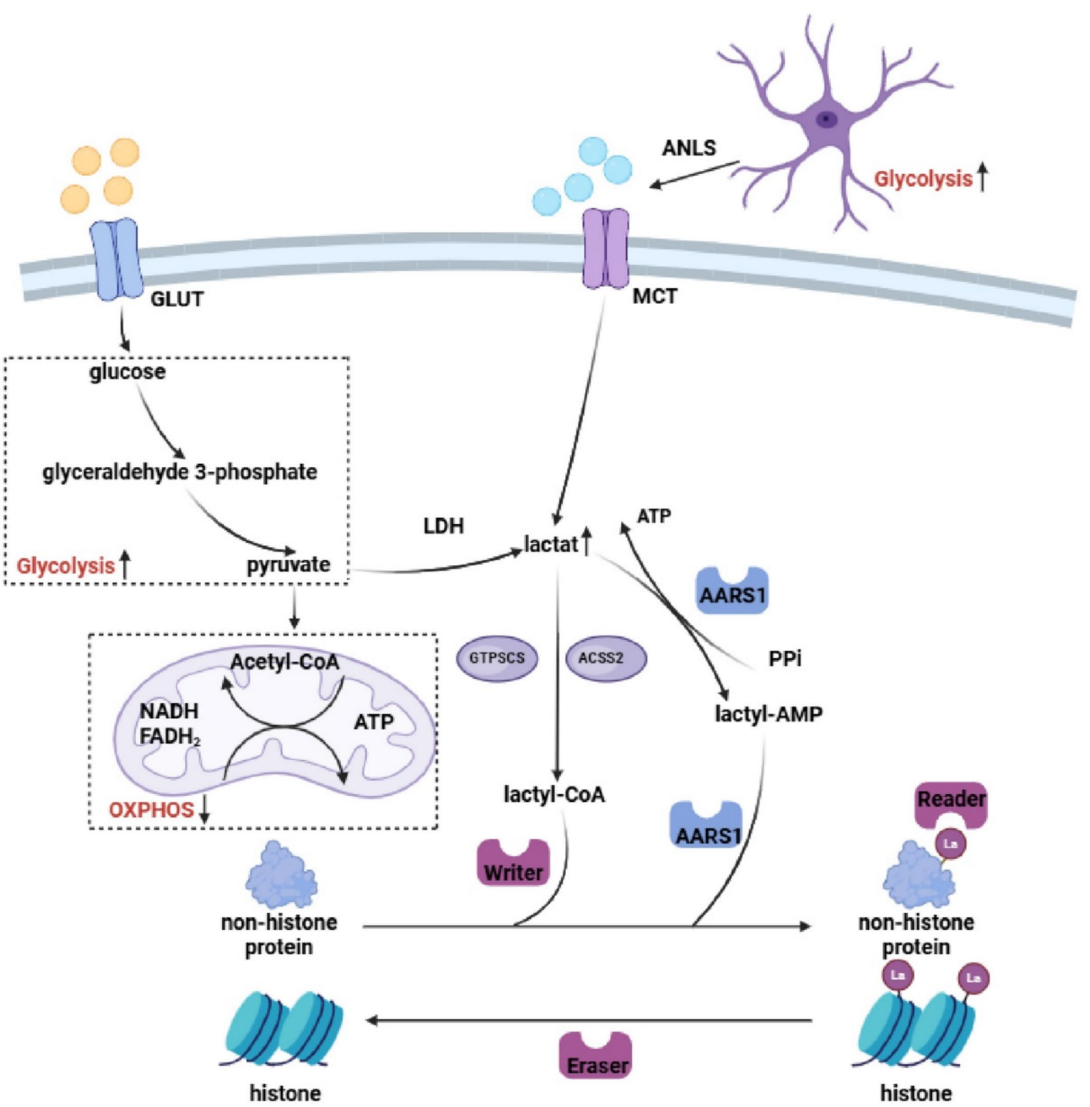
Protein lactylation driven by dysregulated glucose metabolism in AD. Impaired glucose metabolism in the AD brain manifests as mitochondrial dysfunction coupled with enhanced glycolysis, leading to elevated lactate production. Concurrently, glial cells supply abundant lactate via the ANLS, providing a rich substrate for aberrant lactylation. Lactate is converted into lactyl-CoA and subsequently incorporated into proteins through the coordinated action of the “writer–eraser–reader” regulatory machinery.

Accordingly, the dynamic equilibrium of cerebral lactate metabolism constitutes a foundational framework for understanding both the physiological roles and pathological consequences of protein lactylation. Disruption of this homeostasis represents an upstream driver of aberrant lactylation in AD, directly linking energetic crisis to downstream alterations in gene expression programs and cellular responses (Figure 1).

## 3 Molecular logic of protein lactylation

### 3.1 Protein lactylation: a paradigm shift

Protein lactylation is a recently identified post-translational modification that fundamentally revises the long-standing view of lactate as a passive metabolic by-product. In a landmark 2019 study, a lysine modification exhibiting a characteristic mass shift of 72.021 Da was identified on histones, with its abundance tightly coupled to intracellular lactate levels (Zhang et al., 2019). This finding established a direct molecular conduit through which metabolic state can be transduced into epigenetic regulation, thereby redefining lactate as an active signaling metabolite capable of shaping the chromatin landscape.

Lactate generated through glycolysis or imported from the extracellular milieu can conjugate with coenzyme A to form lactyl–coenzyme A, which serves as the direct substrate for protein lactylation (Zhang et al., 2019). This activated metabolite transfers the lactyl moiety to lysine residues on target proteins and is subsequently released. Similar to other post-translational modifications (PTMs), protein lactylation is dynamically governed by a canonical “writers–erasers–readers” regulatory triad, with its activity markedly enhanced under conditions of hypoxia, inflammation, or metabolic stress (Mao et al., 2024; Shi H. et al., 2025; Figure 1; Table 1).

**Table 1:** Key regulatory enzymes mediating protein lactylation.

Category	Representative members	Function	Refs.
Writers	p300 (KAT3B), CBP (KAT3A), GCN5 (KAT2A), TIP60 (KAT5), HBO1 (KAT7), MOF (KAT8)	Catalyze lysine lactylation on target proteins using lactyl-CoA as the donor substrate.	Zhang et al. (2019) and Ren et al. (2025)
	AARS1	Utilizes lactyl-AMP as a substrate to transfer lactyl groups onto protein lysine residues.	Han et al. (2025)
Erasers	HDAC1, HDAC2, HDAC3; SIRT1, SIRT2, SIRT3	Principal de-lactylases responsible for removing lactyl moieties, dynamically regulating protein lactylation.	Moreno-Yruela et al. (2022)
Readers	DPF2, BRG1, TRIM33	Specifically recognize and bind histone lactylation marks, recruiting transcriptional complexes to modulate gene expression.	Hu et al. (2024), Nunez et al. (2024) and Zhai et al. (2024)

The “writing” of lactylation refers to the enzymatic installation of lactyl groups onto lysine residues using lactate and ATP. Lysine acyltransferases (KATs), traditionally characterized as acetyltransferases utilizing acetyl–CoA, have been shown to exhibit lactyltransferase activity. Notably, KAT3B (p300) was initially demonstrated to catalyze lysine lactylation using synthetic lactyl–CoA (Zhang et al., 2019). Genetic ablation of p300 in cells results in a pronounced impairment of lactate-induced histone lactylation (Deng et al., 2025; Zhang et al., 2019). Subsequent studies have expanded this repertoire, revealing that members of all three KAT families—including KAT2A (GCN5), KAT3A (CBP), KAT5 (TIP60), KAT7 (HBO1), and KAT8 (MOF)—possess intrinsic lactylation activity (Ren et al., 2025). In addition, alanyl-tRNA synthetase 1 (AARS1) has been identified as an unconventional lactylation writer, capable of recognizing lactate and converting it into lactyl–AMP, which then donates the lactyl group to lysine residues (Han et al., 2025). The “erasing” of lactylation entails the enzymatic removal of lactyl groups from modified proteins, thereby restoring them to their unmodified state. This process is mediated by lysine deacylases (KDACs), which are broadly categorized into Zn^2+^-dependent histone deacetylases (HDACs) and NAD^+^-dependent sirtuins (SIRTs) (Ren et al., 2025). Several deacetylases have been shown to efficiently catalyze delactylation, with HDAC1–3 and SIRT1–3 displaying robust delactylase activity; among these, HDAC1–3 appear to function as the principal delactylases in cells (Moreno-Yruela et al., 2022). The “reading” of lactylation is executed by effector proteins that selectively recognize and bind lactylated lysine residues, thereby translating this PTM into downstream chromatin and transcriptional outcomes. Identified lactylation readers include DPF2, BRG1, and TRIM33, which specifically recognize lactyl marks and recruit transcriptional complexes to propagate lactylation-dependent signaling in contexts such as tumor biology and immune responses (Hu et al., 2024; Nunez et al., 2024; Zhai et al., 2024). However, the repertoire and functional diversity of lactylation readers remain incompletely defined, underscoring a critical area for future investigation.

Protein lactylation exerts pleiotropic functions across diverse biological contexts, with particularly prominent roles in the nervous system, where metabolic dynamics and cellular plasticity are tightly intertwined. During neurogenesis, lactylation promotes neural cell maturation and lineage specification, thereby supporting the orderly progression of neural development (Xu W. et al., 2025). Beyond development, lactylation participates in activity-dependent signaling to maintain brain homeostasis (Hagihara et al., 2021). Under pathological conditions, this modification emerges as a regulator of neuroinflammation and glial activation, reshaping cellular responses to metabolic stress. Outside the nervous system, lactylation also operates within the tumor microenvironment, where it reprograms immune cell function—including macrophages and T cells—to favor immunosuppressive states and facilitate tumor immune evasion (Certo et al., 2021; Wang Y. et al., 2024). Collectively, these observations position protein lactylation as a broadly conserved metabolic signaling mechanism that integrates metabolic state with context-dependent gene regulation across multiple disease settings.

### 3.2 Histone and non-histone lactylation: distinct mechanisms and functional specialization

**Figure 2:**
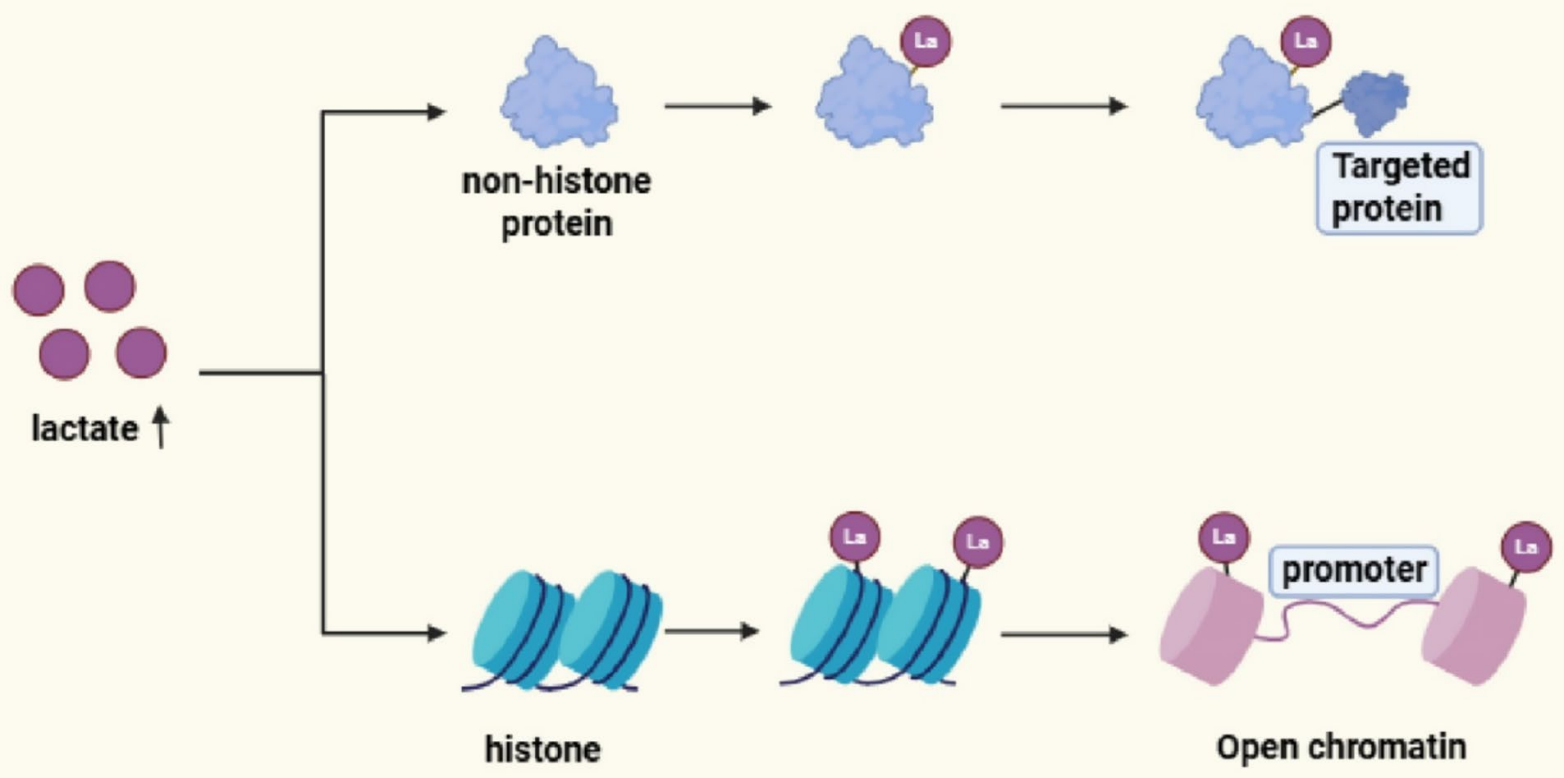
Distinct regulatory logics of histone and non-histone lactylation. Elevated intracellular lactate promotes histone lactylation, which remodels chromatin accessibility and reprograms gene expression through epigenetic mechanisms. In contrast, non-histone lactylation directly modulates protein function by altering the conformation, interaction capacity, stability, or enzymatic activity of downstream target proteins, thereby enabling rapid and context-dependent regulation of cellular processes.

Protein lactylation can be broadly categorized into histone and non-histone modifications based on the identity of their substrates. Rather than representing redundant regulatory layers, these two forms of lactylation operate through distinct molecular logics and temporal scales, together constituting an integrated regulatory network through which lactate governs gene expression, protein function, and ultimately cell fate decisions (Figure 2).

#### 3.2.1 Histone lactylation: transducing metabolic cues into epigenetic information

Histone lactylation was the first characterized form of lactylation and provides a direct mechanistic bridge between intracellular metabolic state and chromatin-based gene regulation (Zhang et al., 2019). As intracellular lactate accumulates, lactylation is deposited at specific lysine residues on core histones—most prominently on H3 and H4, including sites such as H3K18la and H4K12la. These modifications remodel chromatin architecture by partially neutralizing the positive charges on histone tails, thereby weakening histone–DNA interactions and promoting a more permissive chromatin state (Hao et al., 2024). Beyond altering chromatin accessibility, histone lactylation also functions as a molecular signal for the recruitment of dedicated “reader” proteins, including bromodomain-containing factors, which in turn assemble transcriptional activation or repression complexes at defined genomic loci (Shi L. et al., 2025). Through these combined structural and signaling roles, histone lactylation converts metabolic perturbations—such as hypoxia, inflammation, or enhanced glycolytic flux—into sustained epigenetic programs that shape transcriptional output and cellular identity.

#### 3.2.2 Non-histone lactylation: direct and rapid modulation of cellular machinery

In contrast to histone lactylation, non-histone lactylation acts primarily at the level of protein function and signal execution, providing a rapid means for lactate to remodel cellular behavior. Although characterized more recently, non-histone lactylation has been identified on a wide spectrum of proteins, including metabolic enzymes, transcription factors, and DNA damage repair proteins, underscoring its broad regulatory reach (Peng and Du, 2025). Mechanistically, lactylation can directly modulate enzymatic activity by altering charge distribution or steric constraints at functionally critical lysine residues. For example, lactylation of PKM2 at K62 suppresses its catalytic activity, thereby reshaping glycolytic flux (Wang et al., 2017). In addition, lactylation influences protein–protein interactions, as exemplified by lactylation of moesin at K72, which enhances its association with the transforming growth factor-β type I receptor through stabilized hydrogen bonding (Gu et al., 2022). Non-histone lactylation also regulates protein stability through crosstalk with other post-translational modifications. Lactylation of APOC2 at K70 antagonizes ubiquitination, protecting the protein from proteasomal degradation, whereas lactylation of ALDOA at K147 induces conformational changes that enhance resistance to denaturation and turnover (Chen et al., 2024; Shao et al., 2025). Through these mechanisms, non-histone lactylation enables lactate to exert fast, reversible, and highly context-dependent control over core cellular processes, including metabolic reprogramming and signal transduction.

### 3.3 Detection of protein lactylation

Protein lactylation is tightly coupled to intracellular lactate abundance, and fluctuations in lactate levels can therefore serve as an indirect indicator of lactylation dynamics. Lactate concentrations are commonly quantified using commercial lactate assay kits, which typically employ colorimetric methods to measure lactate levels in cells, blood, or tissue homogenates. In addition, enzyme-linked immunosorbent assays (ELISAs) have been applied to assess lactate content in biological samples. For example, colorimetric assays have revealed reduced extracellular lactate levels in astrocytes derived from sporadic and familial AD (sAD/fAD) patients (Bell et al., 2025), whereas ELISA-based measurements have demonstrated elevated CSF lactate concentrations in individuals with AD compared with healthy controls (Zebhauser et al., 2022). Collectively, these approaches provide valuable insight into the relationship between lactate metabolism and AD progression.

Direct detection of protein lactylation relies on the availability of specific antibodies, including pan-lactylation (Pan-Kla) antibodies and site-specific antibodies targeting defined residues such as H4K8la and H4K12la. These reagents enable investigations ranging from global profiling to site-resolved functional analyses. Pan-Kla and site-specific antibodies have been widely applied in western blotting, immunofluorescence, and immunohistochemistry assays. For instance, western blot analyses have shown a marked increase in Pan-Kla signals in senescent microglia and hippocampal tissues from naturally aged mice, with a predominant band observed at approximately 15 kDa (Wei et al., 2023). Subsequent immunoblotting using antibodies against specific histone lactylation sites—including H4K8la, H4K12la, H3K14la, and H3K18la—has further delineated the patterns of histone lactylation alterations in these contexts (Wei et al., 2023).

Mass spectrometry (MS), which separates ions according to their mass-to-charge ratios and determines their precise molecular masses, has emerged as a gold-standard technique for the unbiased, comprehensive, and accurate identification of post-translational modifications, including lactylation (Noberini and Bonaldi, 2023). Liquid chromatography–tandem mass spectrometry (LC–MS/MS) is particularly well suited for this purpose. The initial discovery of histone lactylation was achieved by Zhang et al. (2019) through HPLC–MS/MS analysis of tryptic peptides, in which lysine residues within three specific peptides exhibited a characteristic mass increase of 72.021 Da, providing definitive evidence for histone lactylation. In AD models, MS-based approaches have similarly identified lactylation on amyloid precursor protein (APP) and tau, implicating this modification in the modulation of AD pathology (Tian et al., 2025; Zhang X. et al., 2025). During MS/MS fragmentation, the formation of cyclic immonium ions derived from lactylated lysine further facilitates reliable lactylation detection and the discovery of novel modification sites (Wan et al., 2022).

Fluorescent probe–based strategies have also been developed to interrogate lactylation dynamics. Using this approach, a p-H4K16la–NBD probe has been engineered to enable direct fluorescence-based monitoring of delactylation processes (Fan et al., 2023). However, the specificity and sensitivity of such molecular and fluorescent probes for large-scale lactylation profiling and site identification remain to be rigorously validated.

Beyond biochemical detection, the identification of lactylation-associated genes can be enhanced by integrating single-cell RNA sequencing (scRNA-seq) with bulk transcriptomic analyses, coupled with machine learning–based approaches to prioritize lactylation-related hub genes. In parallel, artificial intelligence (AI)–driven analysis of protein sequences has facilitated rapid and efficient prediction of potential lactylation sites, thereby streamlining downstream experimental validation. A growing suite of computational tools—including FSL-Kla, Deep-Kla, Auto-Kla, Hybrid-Kla, PBert-Kla, DeepKlapred, ABFF-Kla, and EBFF-Kla—has been developed for this purpose (Guan et al., 2024; Jiang et al., 2021; Lai and Gao, 2023, Lai et al., 2025; Lv et al., 2022; Ning et al., 2025; Yang Y. et al., 2024). Together, these methodological advances have substantially accelerated lactylation research, and continued technological innovation is expected to yield increasingly robust and high-throughput strategies for lactylation detection in the future.

## 4 Cell type–specific roles of lactylation in core AD pathology

Building upon advances in lactylation detection, elucidating its precise pathological functions in AD is of paramount importance. Accumulating evidence indicates that protein lactylation is deeply integrated into the core pathogenic cascades of AD. Its impact extends beyond the direct modulation of key neuronal pathogenic proteins, such as APP and tau, to encompass profound metabolic and functional reprogramming of diverse cell populations within the neuroimmune microenvironment, including microglia and astrocytes (Table 2).

**Table 2:** Roles of protein lactylation in core pathological processes of AD.

Target cell	Specific effects & mechanisms	Effect	Refs.
Neurons	APP-K612 lactylation: inhibits APP–BACE1 interaction, promoting degradation via the endosome–lysosome pathway	Reduces Aβ production, improves learning and memory	Tian et al. (2025)
	EPB41L4A-AS1 downregulation: diminishes histone lactylation-mediated regulation of autophagy genes	Attenuates Aβ-induced neuronal apoptosis and synaptic dysfunction	Wang Z. et al. (2024)
	IGF2BP3-K76 lactylation: modulates NRF2 expression, reducing ROS accumulation	Indirectly decreases Aβ deposition	Lu et al. (2024)
	H3K18la elevation: stimulates PSMD14 upregulation, activating PINK1-mediated mitophagy	Mitigates Aβ-induced neuronal apoptosis and synaptic deficits	Xu L. et al. (2025)
	H4K12la: activates FOXO1/PGC-1α pathway, enhancing mitochondrial oxidative stress	Indirectly promotes Aβ accumulation	Yang et al. (2025)
	Tau-K331 lactylation: catalyzed by p300, promotes Tau phosphorylation and cleavage while inhibiting ubiquitin-mediated degradation	Exacerbates pathological Tau aggregation and neurotoxicity	Zhang X. et al. (2025)
	Tau-K677 lactylation: triggers MAPK pathway activation, facilitating ferroptosis	Leads to neuronal injury	An et al. (2024)
	IDH3β downregulation–lactate accumulation: establishes “IDH3β–lactate–PAX6–IDH3β” positive feedback, elevating histone lactylation and Tau hyperphosphorylation	Drives synaptic damage and cognitive deficits	Wang X. et al. (2024) and Zhang et al. (2021)
Microglia	H4K12la elevation: enriched at glycolytic gene promoters (e.g., PKM2), forming a glycolysis–H4K12la–PKM2 positive feedback loop	Enhances pro-inflammatory phenotype, exacerbates neuroinflammation	Pan et al. (2022)
	H4K12la elevation: Activates NLRP3 transcription, affecting mTOR-regulated autophagy and microglial activation	Promotes the accumulation of Aβ plaques, exacerbates neuroinflammation	Wang H. et al. (2025)
	H3K18la elevation: amplifies NF-κB signaling, upregulating SASP factors (e.g., IL-6, IL-8)	Promotes microglial senescence-associated inflammation	Wei et al. (2023)
	YY1-K183 lactylation: regulates transcription of inflammatory genes	Facilitates microglial activation, proliferation, and migration	Huang et al. (2024)
Astrocyte	Lactate-driven Kla: astrocyte-derived lactate is a key driver of neuronal Kla	Induces neuronal death and A1 astrocyte activation	Xiong et al. (2024)
	Reduced LRP1 expression: Enhanced emulsification of ARF1	Inhibits release and transfer of healthy mitochondria to neurons	Stanca et al. (2023) and Zhou et al. (2024)
	Sox9–HK1 axis: injury-induced Sox9 phosphorylation drives HK1-mediated hyper-glycolysis and lactate production, inducing H3K9la at pro-inflammatory gene promoters	Suggests a mechanism promoting neuroinflammation and neurotoxicity in AD	Chen Y. et al. (2025a)

### 4.1 Lactylation-mediated regulation of core pathological proteins in neurons

Neurons constitute the primary cellular locus for Aβ deposition and tau neurofibrillary tangle formation in AD. Lactylation has emerged as a critical regulatory mechanism that directly or indirectly governs the metabolism and toxicity of both pathological proteins, thereby exerting a decisive influence on neuronal fate.

#### 4.1.1 Regulation of APP metabolism and aβ pathology

Aberrant accumulation of Aβ represents a canonical pathological hallmark of AD and originates from dysregulated processing of its precursor, APP. APP is a ubiquitously expressed type I transmembrane non-histone protein that is particularly enriched in neurons. Under pathological AD conditions, APP processing undergoes a fundamental shift toward the amyloidogenic pathway, wherein sequential cleavage by β-site APP-cleaving enzyme 1 (BACE1) and the γ-secretase complex releases Aβ peptides predominantly composed of 40 or 42 amino acids (Al-Kuraishy et al., 2023). Among these, Aβ42—distinguished by two additional hydrophobic residues at its C-terminus—exhibits markedly enhanced hydrophobicity, rendering it more prone to conformational instability and aggregation and establishing it as a principal neurotoxic species driving AD pathology (Lendel et al., 2014; Sharma et al., 2022).

Aβ species compromise neuronal function through multiple direct and indirect mechanisms. They can insert into neuronal membranes, disrupt membrane integrity, and perturb calcium homeostasis, thereby triggering excitotoxicity (Pensalfini et al., 2022). In parallel, Aβ aberrantly interacts with neuronal surface receptors, including NMDA receptors and cellular prion protein, interfering with synaptic signaling (Stoner et al., 2023; Wiatrak et al., 2021). These interactions activate downstream kinase cascades, such as GSK-3β and CDK5, promoting pathological tau hyperphosphorylation. In addition, Aβ induces mitochondrial dysfunction, elevates reactive oxygen species (ROS) production, and precipitates oxidative stress–mediated neuronal apoptosis (Kumari et al., 2023; Massaro et al., 2025). Furthermore, Aβ activates microglia and astrocytes, amplifying neuroinflammatory responses and accelerating disease progression (Ferrari-Souza et al., 2025).

Emerging evidence suggests that lactylation exerts a modulatory influence on Aβ biogenesis. In APP23/PS45 double-transgenic mice, lactylation of APP at lysine 612 (K612) suppresses its interaction with BACE1 and subsequent amyloidogenic cleavage. Concurrently, APP lactylation enhances its association with CD2AP, facilitating endosomal–lysosomal degradation and thereby reducing Aβ production, ultimately improving spatial learning and memory performance. Notably, APP lactylation is diminished in AD models, potentially contributing to pathological Aβ accumulation (Tian et al., 2025). In addition, the long noncoding RNA EPB41L4A-AS1 promotes Aβ clearance by modulating histone lactylation enrichment and transcriptional activation of autophagy-related genes, including ATG3, ATG5, and ATG16L1. In AD, reduced EPB41L4A-AS1 expression compromises this protective mechanism, impairing Aβ clearance (Wang Z. et al., 2024).

Lactylation may also indirectly influence Aβ pathology by regulating oxidative stress. Oxidative stress is a well-established driver of Aβ accumulation and is closely linked to diminished expression of antioxidant defenses (D'Alessandro et al., 2025). Lactylation of non-histone IGF2BP3 has been shown to enhance NRF2 expression, thereby attenuating ROS accumulation and suppressing oxidative stress–induced Aβ deposition (Lu et al., 2024). Moreover, histone lactylation may upregulate PSMD14, activate PINK1-dependent mitophagy, eliminate damaged mitochondria, and preserve mitochondrial homeostasis, collectively mitigating Aβ-induced neuronal apoptosis and synaptic dysfunction (Xu L. et al., 2025). Conversely, elevated H4K12 lactylation has been reported to enhance FOXO1 promoter binding, activate the FOXO1/PGC-1α signaling axis, and exacerbate mitochondrial oxidative stress, indirectly promoting Aβ accumulation (Yang et al., 2025). Neuroinflammation further accelerates Aβ deposition, and lactylation has been implicated as a pro-inflammatory modulator, suggesting that targeting cerebral lactylation may alleviate inflammation-associated Aβ pathology (Fang et al., 2024; Twarowski and Herbet, 2023). Although these findings may appear contradictory, they collectively underscore the dynamic and context-dependent complexity of lactylation-mediated regulation of Aβ pathology.

#### 4.1.2 Lactylation-driven tau pathology

Tau is a microtubule-associated non-histone protein predominantly localized to neuronal axons, where it stabilizes microtubules and supports cytoskeletal integrity and axonal transport (Parra Bravo et al., 2024). In AD, tau undergoes a spectrum of aberrant post-translational modifications, among which pathological hyperphosphorylation is considered a central pathogenic event. Phosphorylated tau species are key biomarkers for early AD diagnosis and are detectable in cerebrospinal fluid and blood (Wojdała et al., 2025). In AD brains, heightened activity of kinases such as GSK-3β and CDK5, coupled with reduced activity of phosphatases such as PP2A, drives tau phosphorylation beyond physiological thresholds (Schweiger et al., 2017). Hyperphosphorylated tau dissociates from microtubules, undergoes misfolding and aggregation, and ultimately forms neurofibrillary tangles (NFTs). NFTs serve as terminal pathological effectors, destabilizing microtubules, impairing axonal transport, and precipitating synaptic failure—structural substrates of early cognitive decline (Jiang et al., 2025). Tau oligomers themselves exert direct neurotoxicity, promote neuroinflammation, induce cellular senescence, and accelerate disease progression (Gaikwad et al., 2024).

Beyond phosphorylation, tau undergoes multiple additional post-translational modifications, including acetylation and ubiquitination; however, their pathological relevance in AD is not equivalent (Haj-Yahya and Lashuel, 2018; Jiang et al., 2025). Lactylation modifies tau by attaching lactyl groups to specific lysine residues, thereby altering its charge properties and conformational landscape. Tau lactylation levels are markedly elevated in AD brains and have been shown to promote tau phosphorylation and proteolytic cleavage, exacerbating pathological tau accumulation. Among identified sites, lysine 331 (K331) is particularly prominent; its lactylation is catalyzed by the acetyltransferase p300, is directly inducible by lactate, and depends on endogenous lactate production mediated by lactate dehydrogenase A (LDHA) (Zhang X. et al., 2025). In addition, lactylation at tau K677 has been reported to activate MAPK signaling, promote ferroptosis, and culminate in neuronal injury (An et al., 2024). In AD, downregulation of isocitrate dehydrogenase 3β (IDH3β) induces mitochondrial dysfunction and lactate accumulation, thereby enhancing tau hyperphosphorylation and aggravating cognitive deficits (Wang X. et al., 2024; Zhang et al., 2021). Lactate, serving as a lactyl donor, promotes histone lactylation—particularly at H4K12 and H3K18—thereby upregulating paired box gene 6 (PAX6). As a transcriptional repressor of IDH3β, PAX6 further suppresses IDH3β expression, establishing a positive feedback loop (“IDH3β–lactate–PAX6–IDH3β”) that ultimately drives tau hyperphosphorylation, synaptic injury, and learning and memory impairment (Wang X. et al., 2024).

Tau clearance represents another critical regulatory axis, with ubiquitination serving as a principal degradative pathway (Wang L. et al., 2025). Experimental evidence indicates that lactylation suppresses tau polyubiquitination, suggesting a stabilizing effect of lactylation on tau protein. Conversely, LDHA knockdown or expression of a lactylation-resistant tau mutant (tau-3KR) enhances tau ubiquitination, reinforcing the notion that lactylation interferes with normal tau degradation pathways (Zhang X. et al., 2025). Collectively, these findings position lactylation as a potent driver of tau pathology by simultaneously promoting pathological phosphorylation, inhibiting clearance, and amplifying neurotoxicity in AD.

### 4.2 Lactylation in glial cell functional reprogramming

Microglia and astrocytes undergo profound metabolic and functional reprogramming in AD, with lactylation emerging as a pivotal regulatory mechanism. Notably, the modes of action and pathological consequences of lactylation are highly cell type–specific.

#### 4.2.1 Microglia: lactylation-driven metabolic–inflammatory feedforward loops

Microglia are the resident immune cells of the central nervous system, originating from the embryonic yolk sac (Ginhoux et al., 2010). Under physiological conditions, microglia mediate immune surveillance and debris clearance through the expression of genes such as CX3CR1 and P2Y12 (Shimizu and Prinz, 2025). In AD, microglia exhibit pronounced functional heterogeneity. During early disease stages, they exert neuroprotective effects by clearing Aβ, a process critically dependent on triggering receptor expressed on myeloid cells 2 (TREM2), which directly recognizes and facilitates Aβ uptake (Long et al., 2022; Zhao et al., 2018). However, chronic activation drives microglia toward a pro-inflammatory phenotype characterized by the secretion of cytokines such as TNF-α and IL-6, exacerbating synaptic loss (Masuda et al., 2020). Moreover, microglia-derived IL-1β and TNF-α further enhance β-secretase expression, increasing Aβ production and establishing a vicious cycle that accelerates disease progression (Heneka et al., 2015).

Pro-inflammatory microglial activation represents a hallmark response in AD and is accompanied by metabolic reprogramming from oxidative phosphorylation to aerobic glycolysis (Gao et al., 2025). This metabolic shift directly promotes the release of inflammatory mediators, including TNF-α, IL-6, and IL-1β, whereas inhibition of glycolysis has been shown to attenuate AD-associated neuroinflammation (Miao et al., 2023; Pan et al., 2022). Lactate-driven metabolic reprogramming critically shapes microglial energy states and functional phenotypes. Microglial phenotypic switching is largely driven by histone lactylation. In AD mouse models and human brain tissues, microglia localized near Aβ plaques exhibit elevated H4K12 lactylation, which is enriched at promoters of glycolytic genes such as HIF-1α and LDHA, thereby enhancing their transcription and amplifying glycolytic flux (Pan et al., 2022). This establishes a self-reinforcing feedforward loop in which H4K12la upregulates pyruvate kinase M2 (PKM2), PKM2 promotes lactate production, and lactate further drives lactylation, collectively exacerbating glycolytic hyperactivation and metabolic imbalance in the AD brain (Pan et al., 2022).

Elevated H4K12la also activates NLRP3 transcription by modulating mTOR-regulated autophagy and microglial activation, thereby promoting Aβ plaque accumulation (Wang H. et al., 2025). Pharmacological or genetic inhibition of PKM2 dampens microglial activation and improves learning and memory performance in AD mouse models, highlighting disruption of this feedforward loop as a potential therapeutic strategy (Pan et al., 2022). In addition, global lactylation levels are increased in senescent microglia and hippocampal tissues of AD model mice, where H3K18la enhances NF-κB binding to target promoters and upregulates senescence-associated secretory phenotype (SASP) factors such as IL-6 and IL-8 (Wei et al., 2023). Furthermore, lactylation of the non-histone protein YY1 at lysine 183 further promotes microglial activation, proliferation, and migration by regulating transcription of inflammatory genes including STAT3 and CCL5 (Huang et al., 2024).

#### 4.2.2 Astrocytes: lactylation and disrupted metabolic support

Astrocytes are the most abundant glial cell population in the central nervous system and play essential roles in maintaining metabolic homeostasis, synaptic function, and blood–brain barrier integrity (Barros et al., 2024). Under physiological conditions, astrocytes preferentially engage in glycolysis, producing lactate that fuels neurons via the ANLS and contributes to the clearance of pathological substrates such as Aβ (Bolaños and Magistretti, 2025; Park et al., 2024). In AD, astrocytes exhibit dysregulated neuroinflammatory and metabolic signaling pathways, thereby reshaping the cerebral microenvironment and facilitating disease progression (Kim et al., 2024; Smith et al., 2022).

During early AD pathology, astrocytes upregulate glycolytic activity and generate increased amounts of lactate to support neuronal energy demands through the ANLS (Calì et al., 2024). Elevated lactate levels may regulate the expression of glycolytic genes, including PFKFB3 and HK2, via lactylation-dependent mechanisms, thereby influencing astrocytic proliferation and metabolic states (Chen Z. et al., 2025; Xiong et al., 2025). Excessive lactate accumulation in the brain promotes the formation of lactylation marks and induces neuronal death as well as activation of neurotoxic A1 astrocytes (Xiong et al., 2024). Experimental evidence indicates that protein lactylation predominantly occurs in neurons, with astrocyte-derived lactate serving as a critical upstream driver. Astrocyte-specific deletion of LDHA or pharmacological inhibition of p300 with A-485 to block lactylation formation markedly improves neurological recovery (Xiong et al., 2024).

In astrocytes, low-density lipoprotein receptor–related protein 1 (LRP1) suppresses glucose uptake, glycolysis, and lactate production, thereby reducing lactylation of the non-histone ADP-ribosylation factor 1 (ARF1) and promoting the release and transfer of healthy mitochondria to neurons (Stanca et al., 2023). In AD, reduced astrocytic LRP1 expression may exacerbate ARF1 lactylation and accelerate disease progression (Stanca et al., 2023; Zhou et al., 2024). In the context of neuropathic pain, the astrocytic transcription factor Sox9 regulates hexokinase 1 (HK1) expression. Neural injury induces aberrant phosphorylation of Sox9, triggering excessive HK1 activation and driving high-rate glycolysis in astrocytes (Chen Y. et al., 2025a). The resulting lactate surplus induces H3K9 lactylation, remodels promoters of pro-inflammatory and neurotoxic genes, and activates their transcriptional programs (Chen Y. et al., 2025a). Notably, Sox9 overexpression is also observed in AD astrocytes, suggesting that analogous metabolic–epigenetic coupling mechanisms may contribute to AD pathogenesis (Choi et al., 2025).

Collectively, lactate and lactylation profoundly shape microglial and astrocytic phenotypes in AD by coordinating glycolytic metabolism, inflammatory signaling, and intercellular communication, thereby influencing the trajectory of disease progression.

**Figure 3:**
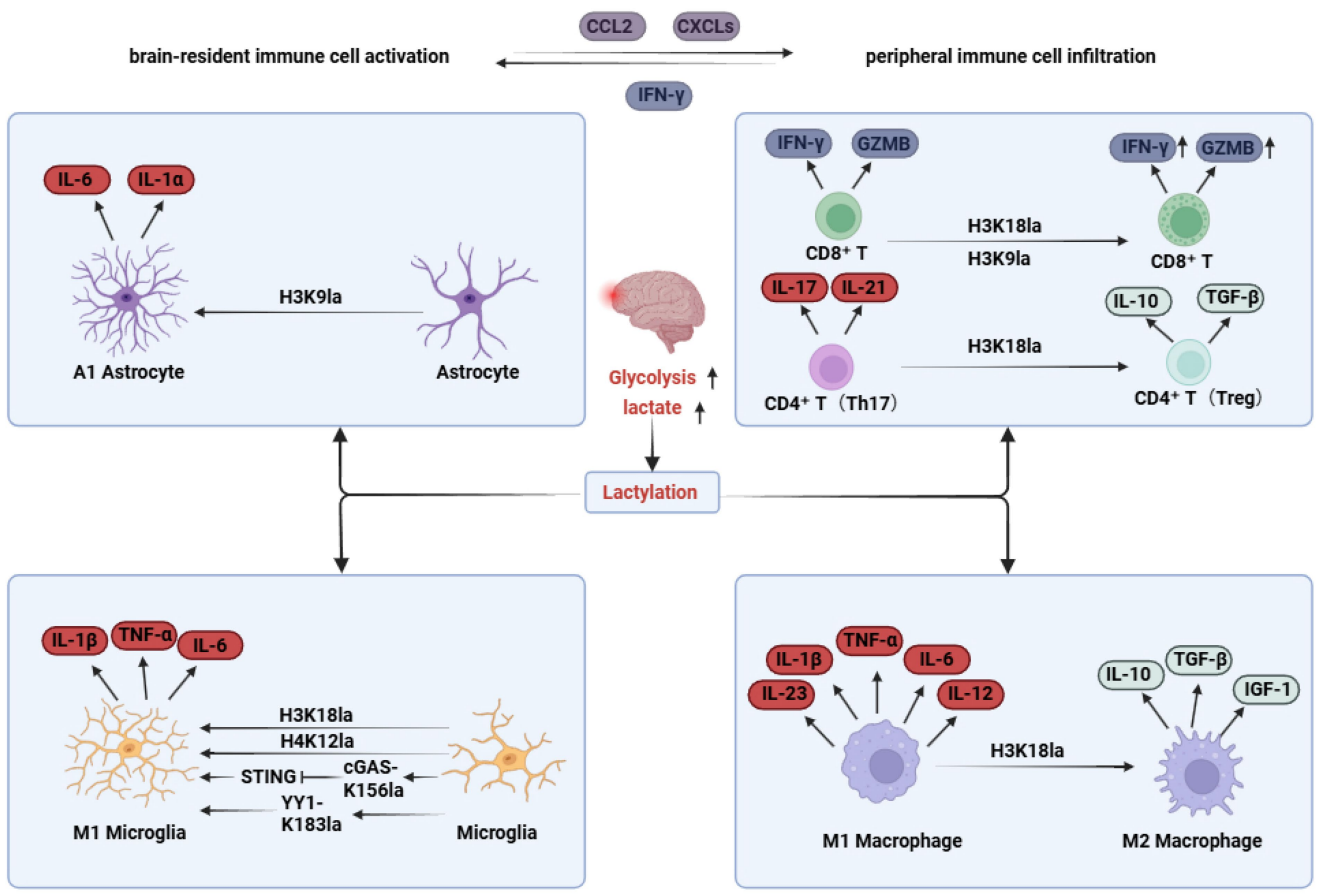
Lactate-centered metabolic–epigenetic–immune crosstalk in AD. Lactate accumulation in the AD brain not only amplifies lactylation within resident CNS immune cells, exacerbating neuroinflammatory responses, but also induces the secretion of chemokines such as CCL2 and CXCLs, promoting infiltration of peripheral immune cells. Within this high-lactate milieu, infiltrating peripheral immune cells undergo lactylation themselves, actively contributing to cerebral immune modulation.

## 5 Lactate as a nexus linking metabolic reprogramming, epigenetic remodeling and immune regulation in AD

Once regarded merely as the terminal by-product of glycolysis, lactate is now recognized as a potent signaling metabolite that exerts pleiotropic regulatory functions through protein lactylation. By doing so, lactate establishes a multidimensional regulatory network that integrates cellular metabolic reprogramming, epigenetic remodeling and immune modulation, thereby occupying a central nodal position in neurodegenerative disorders such as AD (Figure 3). Accumulating evidence indicates that lactate, via histone and non-histone lactylation, directly translates metabolic states into transcriptional outputs, profoundly shaping both innate and adaptive immune landscapes (Fanucchi et al., 2021). Trained immunity, a form of innate immune memory underpinned by metabolic and epigenetic reprogramming, is highly dependent on lactate metabolism and lactylation. For instance, stimulation with β-glucan or Bacillus Calmette–Guérin (BCG) enhances glycolysis in monocytes via the mTOR–HIF-1α axis, leading to lactate accumulation and enrichment of H3K18la at promoters of inflammatory genes, thereby establishing a durable pro-inflammatory transcriptional memory (Su et al., 2022; Ziogas et al., 2025). In the tumor microenvironment, lactate similarly orchestrates intercellular metabolic communication through a “lactate shuttle” mechanism, emerging as a central mediator of immune-cell metabolic reprogramming (Wang et al., 2023). Notably, during trained immunity, monocytes preferentially utilize lactate rather than glucose as a substrate for the tricarboxylic acid (TCA) cycle, while lactate metabolism in CD8^+^ T cells is indispensable for sustaining effective antitumor immunity (Apostolova and Pearce, 2022; Cai et al., 2025).

**Figure 4:**
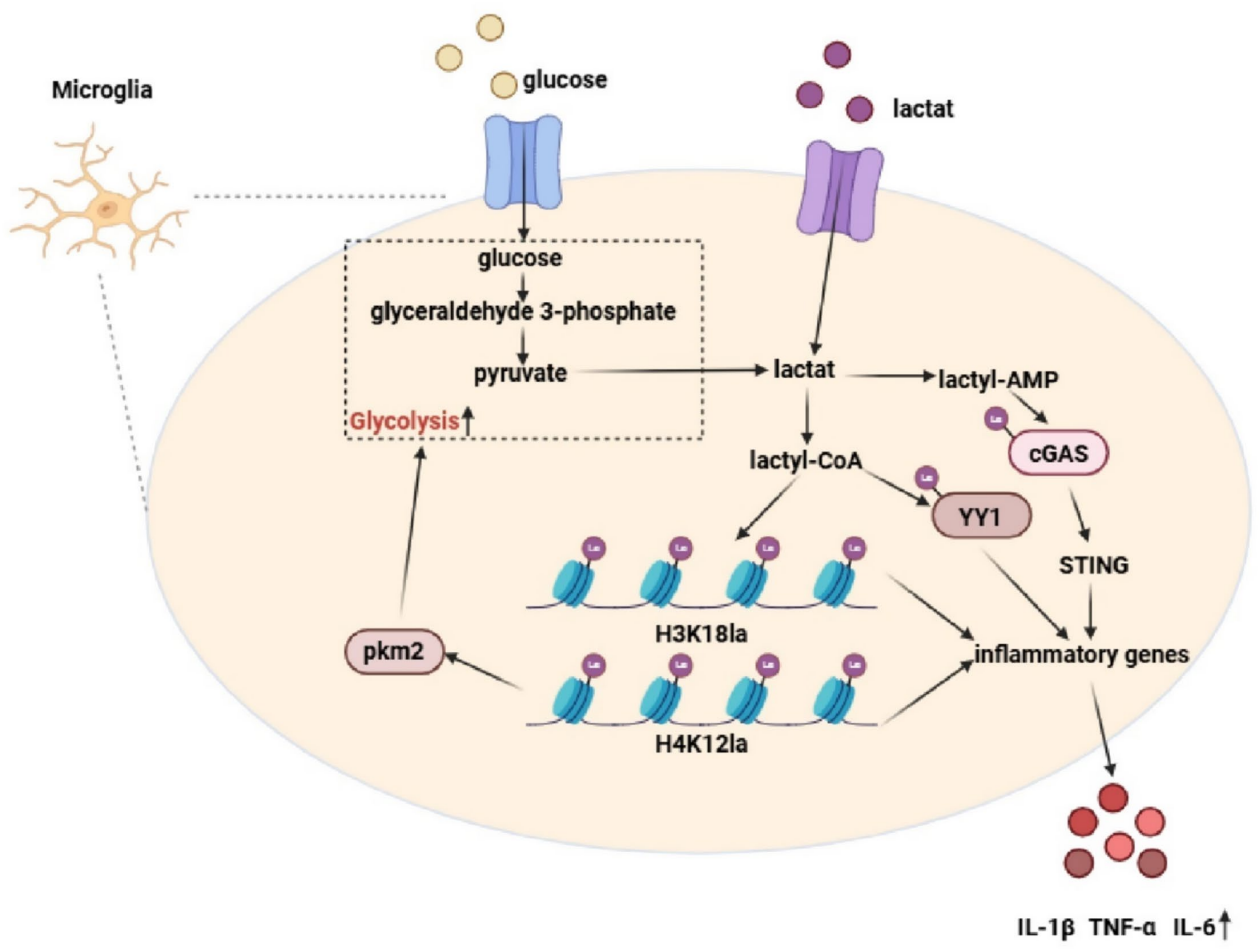
Lactylation-driven inflammatory circuitry in microglia. In AD microglia, elevated intracellular lactate is converted into lactyl-CoA, which fuels both histone lactylation (including H3K18la and H4K12la) and non-histone lactylation of the transcription factor YY1. In parallel, lactate-derived lactyl-AMP activates the cGAS–STING signaling pathway. These epigenetic and innate immune signaling axes converge to transcriptionally induce a broad repertoire of inflammatory genes, resulting in increased expression and secretion of pro-inflammatory cytokines such as IL-1β, TNF-α, and IL-6. Notably, H4K12 lactylation further enhances PKM2 expression, thereby establishing a self-reinforcing “glycolysis–lactylation–inflammation” feedback loop that sustains microglial pro-inflammatory activation in the AD brain.

Lactate-driven metabolic and epigenetic reprogramming markedly enhances immune-cell plasticity, facilitating the establishment of disease-specific immune states (Chen et al., 2021). In the context of AD, lactate accumulation not only acidifies the local microenvironment but also serves as a critical substrate for histone lactylation, directly governing the fate and function of central immune cells. As discussed in Section 4, aberrantly elevated lactylation in microglia and astrocytes promotes their polarization toward pro-inflammatory phenotypes, thereby exacerbating neuroinflammatory responses (Chen Y. et al., 2025a; Pan et al., 2022; Sofroniew, 2024; Figure 4). In addition, lactylation of the innate immune DNA sensor cyclic GMP–AMP synthase (cGAS) in microglia may modulate ligand recognition and signal activation, fine-tuning the activity of the cGAS–STING–NLRP3 inflammatory axis during AD progression (Li et al., 2024). With the onset of blood–brain barrier dysfunction and the release of chemokines (such as CCL2 and CXCLs) by activated glial cells, peripheral immune cells progressively infiltrate the central nervous system and contribute to disease evolution (Balkhi et al., 2025; Brigas et al., 2021; Cao et al., 2025). Among these, infiltrating CD8^+^ T cells activate microglia through the secretion of cytotoxic mediators, inducing the release of pro-inflammatory cytokines including IFN-γ and IL-1β, thereby amplifying neuroinflammation and accelerating Aβ pathology (van Olst et al., 2022; Zhang S. et al., 2025a). Strikingly, highly activated CD8^+^ T cells exhibit markedly elevated levels of H3K18la and H3K9la, which positively correlate with disease severity; pharmacological inhibition of LDHA reduces histone lactylation and attenuates cytotoxic effector functions (Raychaudhuri et al., 2024). Within the CD4^+^ T-cell compartment, Th1 and Th17 subsets secrete IFN-γ, TNF-α and IL-17, directly or indirectly promoting the polarization of microglia toward an M1-like phenotype and thereby aggravating neuroinflammation and neurodegeneration (Machhi et al., 2021). Conversely, Aβ-induced Th2 cells suppress IFN-γ production in Th1 and Th17 cells, downregulate CD86 and CD40 expression in microglia, and ultimately mitigate inflammatory responses (McQuillan et al., 2010). Although lactate has been shown to drive Th17 differentiation toward a pro-inflammatory phenotype, extracellular lactate can paradoxically reprogram Th17 cells into immunosuppressive, Treg-like cells by reshaping their metabolic and epigenetic states (Certo et al., 2021; Lopez Krol et al., 2022). In addition, macrophages residing at central nervous system interfaces actively participate in immune regulation during AD. Activation of peripheral M1 macrophages can trigger NLRP3 inflammasome cascades, releasing IL-1β and IL-18 to further potentiate pro-inflammatory microglial activation and drive neuroinflammatory progression (Cheon et al., 2025; Wen et al., 2024). Consistent with this notion, multiple studies have reported an increased proportion of M1 macrophages in patients with AD (Hsieh et al., 2020; Zhao et al., 2023). Notably, H3K18la has emerged as a key determinant of macrophage metabolic adaptation, modulating not only the magnitude of inflammatory responses but also enhancing phenotypic plasticity and facilitating transitions toward reparative states (Bao et al., 2025).

Collectively, these findings converge on a central concept: lactate, through lactylation-dependent mechanisms, serves as a critical molecular bridge linking metabolism, epigenetic regulation and immune control. This metabolic–epigenetic–immune axis not only reshapes the function and memory of central and peripheral immune cells but also plays a decisive role in the initiation and progression of AD, thereby providing a compelling theoretical framework and therapeutic opportunity for targeting lactate metabolism and lactylation pathways.

## 6 Crosstalk between lactylation and other post-translational modifications

Lactylation engages in intricate interplay with other post-translational modifications (PTMs), including acetylation, methylation, ubiquitination, and phosphorylation, collectively orchestrating protein function and signaling networks (Hai et al., 2025). Such interactions occur through competition for shared lysine residues, utilization of overlapping enzymatic machineries, cascade signaling, or conformational modulation, forming a complex regulatory landscape that finely tunes cellular processes in response to metabolic fluctuations.

Among these, the crosstalk between lactylation and acetylation is particularly pronounced, reflecting deep coupling at the levels of metabolic origin, enzymatic machinery, and site occupancy. Both lactylation and acetylation derive from glycolytic pyruvate: lactate serves as the donor for lactylation, while acetyl-CoA fuels acetylation (Zebhauser et al., 2022; Zhang et al., 2019). Under hypoxic conditions, pyruvate is preferentially converted to lactate, elevating lactylation while diminishing acetylation; conversely, aerobic conditions favor acetylation (Ren et al., 2025). This metabolic partitioning directly reshapes the histone modification landscape. Shared “writer” and “eraser” systems further exacerbate competitive occupancy; for instance, histone H3K18 in hepatic stellate cells exhibits direct competition between lactylation and acetylation (Rho et al., 2023). Beyond acetylation, lactylation also interfaces with crotonylation, succinylation, and phosphorylation. In crotonylation, H3K9cr and H3K18la co-localize extensively within active chromatin regions, and HDACs can concurrently remove both modifications, implying coordinated regulatory mechanisms (Dai et al., 2022). Succinylation and lactylation can compete for identical residues: K311 succinylation of PKM2 promotes its nuclear translocation, enhancing HIF-1α–driven lactate production (Wang et al., 2017); in contrast, K62 lactylation of PKM2 inhibits dimerization, boosts pyruvate kinase activity, and reduces nuclear localization, thereby counteracting the Warburg effect (Wang et al., 2022). Lactylation further modulates protein function via phosphorylation. K331 lactylation of tau enhances its phosphorylation and impedes ubiquitin-mediated degradation, facilitating pathological accumulation and cleavage (Zhang X. et al., 2025). Similarly, K150 lactylation of the transcription factor Twist1 promotes its phosphorylation and nuclear translocation, activating TGFB1 transcription and driving a fibrotic phenotype (Xu et al., 2024). Phosphorylation can reciprocally activate acetyltransferase p300, thereby promoting lactylation (Ma Y. et al., 2025). Lactylation and ubiquitination frequently compete for the same lysine residues. For example, K162 lactylation of RHOA inhibits ubiquitin-mediated degradation, increasing protein stability (Ma C. et al., 2025). Likewise, tau K331 lactylation diminishes ubiquitination while augmenting phosphorylation and proteolytic cleavage, with potential pathological relevance in AD (Zhang X. et al., 2025). Furthermore, lactylation intersects with methylation to regulate gene expression; H3K18la enrichment at target promoters remodels chromatin and activates key RNA-modifying regulators—including writers, readers, and erasers—coordinating RNA-dependent control of mRNA stability and translation, and highlighting a prospective therapeutic axis in AD (Liu S. et al., 2025).

Elucidating these layers of crosstalk is critical for devising precise disease-modifying interventions, as targeting a single modification may be insufficient to reverse pathological states. Future studies leveraging multi-omics approaches at single-cell resolution are essential to resolve dynamic PTM landscapes, laying the conceptual groundwork for dual-targeted “metabolic–epigenetic” therapeutic strategies.

## 7 Therapeutic implications, challenges, and future directions

The burgeoning interest in protein lactylation within the context of AD stems from its unique role as a “metabolic–epigenetic” nexus, offering distinct translational advantages over conventional targets. Foremost, lactate—the donor substrate of lactylation—serves as a direct readout of glycolytic flux and oxidative phosphorylation coupling (Schurr, 2023). Early in AD, cerebral glucose hypometabolism coupled with compensatory glycolytic upregulation disrupts lactate homeostasis, rendering lactylation a real-time “molecular gauge” of pathological energy dysregulation (Lu et al., 2025). In this regard, lactylation functions as a real-time indicator of pathological metabolic states rather than a downstream consequence of irreversible degeneration. Compared with multifunctional metabolic intermediates such as acetyl-CoA, which occupy highly interconnected and pleiotropic metabolic networks, lactate is generated within a relatively constrained metabolic context. This biochemical specificity enables lactylation to selectively encode distinct metabolic configurations—most notably aerobic glycolysis—into regulatory signals. Consequently, interventions targeting lactate production, transport, or site-specific lactylation machinery may, in principle, offer a narrower and more controllable therapeutic window than approaches aimed at core metabolic hubs (Ren et al., 2025; Sung et al., 2023; Zhang et al., 2019).

Clinically, protein lactylation research offers fresh avenues for early detection and dynamic monitoring. Histone lactylation–associated genes, including ARID5B, SESN1, and XPA, exhibit biomarker potential, whereas direct assessment of metabolic intermediates and their modified forms provides a more immediate readout (Guo et al., 2024). Elevated lactate in the cerebrospinal fluid (CSF) of early-stage AD patients indicates active lactylation (Zebhauser et al., 2022), and quantifying lactylated proteins—such as tau—could yield real-time markers of intracerebral pathology. Single-cell analyses reveal peripheral monocytes from AD patients display hyperactive glycolysis and aberrant H3K18la, offering systemic metabolic–epigenetic signatures for disease staging (Ramakrishnan et al., 2024).

Translating advances in lactylation biology into clinical application remains a major challenge. A central obstacle lies in the intrinsic complexity of lactylation as a regulatory modification. Lactylation shares core enzymatic machinery—such as p300/CBP and HDACs—with acetylation and other lysine-based post-translational modifications, yet how competitive or cooperative interactions at individual residues shape AD pathogenesis remains largely unresolved. Moreover, the functional consequences of lactylation are highly context dependent, varying with substrate identity, modification site, cell type, and modification stoichiometry, with the relative contribution of each parameter still poorly defined.

Progress is further limited by methodological constraints. Much of the current evidence is derived from post-mortem human tissue or transgenic animal models, providing limited insight into the spatiotemporal dynamics of lactylation *in vivo*. Tools capable of monitoring lactylation in real time, with cell-type and regional resolution, are still lacking. In addition, the distribution and functional divergence of lactylation across neuronal and glial populations, as well as across distinct brain regions, remain insufficiently explored, hindering a comprehensive understanding of its role in selective vulnerability during AD progression.

Nevertheless, accumulating mechanistic insights suggest several potential therapeutic directions worthy of systematic evaluation. One strategy involves modulating pathological lactate production by targeting key glycolytic regulators, such as PDK or PKM2, although the efficacy and safety of this approach in AD models remain to be established (Pan et al., 2022; Sakimura et al., 2024). Alternatively, interference with lactate shuttling through inhibition of monocarboxylate transporters (MCTs) may disrupt maladaptive metabolic coupling between glial cells and neurons; however, such interventions will require precise temporal and cell-type specificity to avoid impairing physiological energy support (Halford et al., 2023). At the level of epigenetic regulation, selective inhibition of acetyltransferases, such as p300/CBP, may mitigate H4K12la-driven microglial dysfunction and tau lactylation (Pan et al., 2022; Zhang X. et al., 2025). Lifestyle interventions, including aerobic exercise, have been shown to modulate cerebral lactate levels and enhance SNAP91 lactylation, thereby stabilizing synaptic architecture (Yan et al., 2024; Zhang S. et al., 2025b). These findings raise the intriguing possibility that the cognitive benefits of lifestyle-based interventions may, at least in part, be mediated through modulation of the metabolic–epigenetic axis.

Future investigations should integrate single-cell proteomics with spatial omics to construct three-dimensional lactylation atlases within the AD brain, unravel cross-talk networks among PTMs, and employ emerging tools such as orthogonal Mb-Pyl Kla-RS/Pyl-tRNA systems for site-specific lactylation manipulation to establish causality (Ma C. et al., 2025). Translationally, cell-type–specific interventions are essential; for instance, selective overexpression of lactylation “eraser” enzymes in neurons versus microglia in AD models could delineate differential pathological and behavioral outcomes, emphasizing the necessity of targeted approaches. Complementary strategies include temporally controlled interventions to navigate lactate’s dualistic effects, development of small-molecule inhibitors against pathogenic lactylation sites (e.g., H4K12la in microglia, K331la in tau), or selective enhancement of protective modifications (e.g., K612la in neuronal APP) via blood–brain barrier–permeable delivery systems. Combined metabolic–epigenetic therapies, such as co-administration of glycolytic and p300 inhibitors, may synergistically suppress lactate production and histone lactylation while mitigating compensatory metabolic responses. Additionally, lactylated proteins in CSF or plasma-derived exosomes may serve as novel diagnostic biomarkers, and AI-driven predictive models could identify disease-relevant modification sites.

By leveraging refined experimental designs and innovative technological platforms to elucidate cell-type–specific mechanisms and dynamic patterns, these efforts promise not only to deepen mechanistic understanding of AD pathology but also to catalyze the development of novel therapeutic strategies targeting the “metabolic–epigenetic” axis, with potential applicability across other neurodegenerative disorders.

## 8 Conclusion

The emergence of protein lactylation as a novel post-translational modification offers a transformative metabolic–epigenetic lens through which to interrogate the pathogenesis of AD. Here, we have systematically delineated the metabolic underpinnings of lactylation within the brain, its dynamic “writer–eraser–reader” regulatory circuitry, and its pivotal involvement in core AD pathologies, including Aβ production, tau aggregation, glial cell activation, and neuroinflammation. Collectively, these findings establish lactylation as a central molecular bridge linking dysregulated energy metabolism, epigenetic remodeling, and impaired immune responses.

As evidence accumulates, however, it has become increasingly clear that the role of lactylation in AD cannot be reduced to a unidirectional pathogenic or protective effect. Instead, lactylation exhibits pronounced bidirectionality that is highly dependent on modification site, cellular context, and disease stage. For example, lactylation of APP at K612 promotes lysosomal degradation and limits Aβ production, whereas histone H4K12 lactylation enhances oxidative stress through activation of the FOXO1–PGC-1α axis, indirectly facilitating amyloid accumulation. Similarly, H4K12 lactylation in microglia reinforces pro-inflammatory feedforward signaling, while H3K18 lactylation in macrophages has been linked to reparative phenotypic transitions. These apparently opposing outcomes underscore a fundamental principle of lactylation biology: distinct lysine residues—even within the same protein—encode divergent functional consequences by reshaping protein conformation, interaction networks, and downstream signaling pathways. Moreover, the regulatory logic of lactylation differs intrinsically among neurons, microglia, and astrocytes, reflecting differences in basal metabolic programs, lactate shuttle efficiency, and effector landscapes.

This contextual dependency further extends across disease progression. Lactylation may initially function as an adaptive or compensatory response to metabolic stress—facilitating protein turnover, inflammatory resolution, or energy redistribution—but prolonged substrate accumulation and impaired clearance mechanisms can progressively convert this response into a driver of pathology. In this sense, lactylation should be viewed not as a static modifier but as a dynamic metabolic sensor whose functional valence shifts along the disease timeline.

Beyond cell type and temporal dynamics, emerging evidence suggests that lactylation is also shaped by regional metabolic heterogeneity within the brain. Metabolic impairment in early AD is not uniform but preferentially affects vulnerable regions such as the hippocampus and association cortex. These regions differ markedly in cellular composition, synaptic activity, and metabolic demand, resulting in distinct lactate dynamics and, consequently, region-specific lactylation landscapes. The concept of “regional metabolic susceptibility” provides a useful framework to interpret these observations, proposing that intrinsic metabolic traits and cellular architecture determine how local metabolic stress is encoded into epigenetic and transcriptional programs. Deciphering how region-specific lactylation patterns intersect with local cell composition, metabolic flux, and selective vulnerability will be essential for understanding why certain neural circuits succumb early in AD.

In summary, protein lactylation in AD is characterized by pronounced spatiotemporal plasticity, cell-type specificity, and regional heterogeneity. While this complexity poses substantial challenges for mechanistic dissection, it also creates opportunities for precision intervention. A deeper understanding of the rules governing lactylation dynamics across defined cellular and regional contexts may ultimately enable the stratification of disease states and the development of targeted metabolic–epigenetic therapies, thereby advancing AD research toward a precision medicine paradigm.
